# Clinical manifestations of wasp stings: a case report and a review of literature

**DOI:** 10.1186/s41182-022-00475-8

**Published:** 2022-10-28

**Authors:** Pramith Ruwanpathirana, Dilshan Priyankara

**Affiliations:** Medical Intensive Unit, National Hospital Sri Lanka, 435/11, Thimbirigasyaya Road, Colombo 05, Sri Lanka

**Keywords:** Case report, Multi-organ, Sting, Toxin, Vespa, Wasp

## Abstract

**Background:**

Wasp stinging, a neglected tropical entity can have a myriad of local and systemic effects. We present a case of multi-organ injury following multiple wasp stings and a review of literature on the systemic manifestations of wasp stings.

**Case presentation:**

A 48-year-old Sri Lankan male who suffered multiple wasp stings, developed an anaphylactic shock with respiratory failure, which was treated with adrenaline and mechanical ventilation. Within the next 2 days the patient developed acute fulminant hepatitis, stage III acute kidney injury, rhabdomyolysis, haemolysis and thrombocytopenia. The patient was treated in the intensive care unit with ionopressors and continuous renal replacement therapy (CRRT). Haemoadsorbant therapy was used in adjunct with CRRT. There was a gradual recovery of the organ functions over the 1st week. However, the patient succumbed to fungal sepsis on the 16th day despite treatment. We conducted a literature review to identify the various clinical manifestations of wasp stinging. Wasp venom contains enzymes, amines, peptides and other compounds. These proteins can cause type 1 hypersensitive reactions ranging from local skin irritation to anaphylactic shock. Furthermore, the toxins can cause direct organ injury or delayed hypersensitivity reactions. The commonly affected organs are the kidneys, liver, and muscles. The effect on the haematological system manifests as coagulopathy and/or cytopenia. The heart, nervous system, lungs, intestines and skin can be affected rarely. Treatment is mainly supportive.

**Conclusion:**

In conclusion, wasp envenomation can result in multi-organ injury and attention should be paid in doing further research and establishing evidence-based treatment practices.

## Background

Wasp, hornet and bee stings have caused a total of 1109 deaths from 2000 to 2017 in the United States with an average of 62 deaths per year which accounts for more fatalities than any other venomous animal (1). Wasps belong to the order Hymenoptera and family Vespidae with more than 25,000 species worldwide. The effects of wasps’ sting on humans may range from mild localized skin reaction to toxin induced multi-organ involvement and death.

Sri Lanka, a tropical country has a high prevalence of insect stings. A study done in the North Western province of Sri Lanka revealed that of all the arthropod stings and bites, 57% were caused by the order Hymenoptera [1]. One study conducted in Base Hospital Deniyaya, identified 322 bee and wasp stings from 2011 to 2013[2]. The insect was identified only in 55 cases of which, 46 (83.6%) were *Apis dorsata* (giant honey-bee), 8 (14.5%) were *Vespa tropica* (greater banded hornet) and one was a *Ropalidia marginata* (paper wasp) sting.

We present a case of multiple wasp stings and a review of the literature on systemic manifestations of wasp envenomation and therapeutic options in the management of multi-organ failure.

## Case presentation

A 48-year-old Sri Lankan male suffered multiple wasp stings (around 70 stings) and was immediately admitted to the local hospital due to syncope. He was hypotensive (90/40 mmHg) on admission and was treated for anaphylactic shock with intramuscular (IM) adrenaline, intravenous (IV) hydrocortisone and IV fluids. Over the next 30 min his oxygen saturation dropped with evidence of bronchospasms. He was intubated and ventilated due to increasing difficulty in breathing and reduced level of consciousness. During the next 6 h he became critically unwell needing escalating doses of IV Noradrenaline (0.5 µg/kg/min) and developed anuric acute kidney injury (AKI). Due to lack of intensive care facilities at the local centre, he was transferred to the medical intensive care unit (ICU), National Hospital of Sri Lanka, for further management. The patient did not have other co-morbidities apart from well controlled hypertension. He was known to have allergies to multiple food items and drugs, but had never had a wasp sting before.

**Fig. 1:**
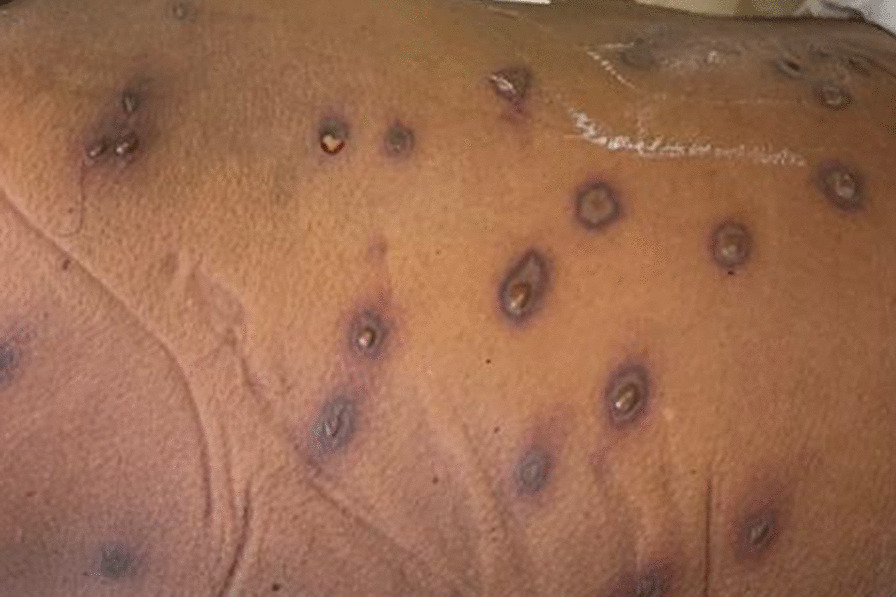
Necrosis and central blistering of the skin surrounding the sites of the stings

Upon admission to the ICU, the patient was volume resuscitated with IV crystalloids and vasopressor support was continued. He was treated with IV antibiotics and steroids in view of anaphylaxis. Over the next 3 days, the patient progressed to acute fulminant hepatic failure, stage III AKI, rhabdomyolysis, coagulopathy, haemolysis and thrombocytopenia (without fragmented red cells in the blood picture). The vasopressor requirement gradually increased to achieve a mean arterial pressure of 65 mmHg and by the 3rd day he was on IV noradrenaline 1 µg/kg/min and IV vasopressin 0.02 U/min. Septic screening was negative. He developed blisters at the site of the stings with surrounding skin necrosis (Fig. 1). The progression of biochemical parameters over the 1st 3 days is given in Table 1.

**Table 1:** Progression of biochemical parameters of organ injury over the 1st 3 days

	On admission 11/10/2021	3rd day of admission 14/10/2021
Liver functions and biochemistries		
AST (5–40 U/L)	680 U/L	18,716 U/L
ALT (7–55 U/L)	1100 U/L	7148 U/L
Alkaline phosphatase (ALP) (44–147 U/L)	453 U/L	223 U/L
Total bilirubin (< 1.2 mg/dL)	10.3 mg/dL	19.7 mg/dL
Direct bilirubin (< 0.4 mg/dL)	4.7 mg/dL	5.0 mg/dL
INR (< 1.1)	1.4	1.84
APTT (21–35 s)	107 s	58.6 s
Albumin (3.4–5.4 g/dL)	3.8 g/dL	2.0 g/dL
Plasma ammonia (10–47 µmol/L)		100.1 µmol/L
Renal functions and biochemistries		
Serum creatinine (S/Cr) (0.74–1.35 mg/dL)	0.49 mg/dL	4.5 mg/dL
Blood urea (6–24 mg/dL)		19.5 mg/dL
Serum sodium (135–145 mEq/L)	136 mEq/L	137 mEq/L
Serum potassium (3.5–5.0 mEq/L)	5.3 mEq/L	6.6 mEq/L
Urine full report		Sugar + 4 Protein + 4 No cells, casts
Urine protein creatinine ratio		U. protein − 166.8 mg/dL U. Creatinine − 4.55 mg/dL UPCR − 36.66
Rhabdomyolysis		
Creatine phosphokinase (CPK) (10–120 µg/)	30,545 µg/L (on 12th /10/ 2021)	43,992 µg/L
Haematological		
Haemoglobin (Hb) (13.8–17.2 g/dL)	18.3 g/dL	13.8 g/dL
White Blood Cells (WBC) (4.5–11.0 × 10^9^/L)	13.9 × 10^9^/L	19.8 × 10^9^/L
Platelets (Plt) (150–400 × 10^9^/L)	280 × 10^9^/L	32 × 10^9^/L
Lung functions		
Ventilatory mode	Volume control ventilation	Volume control ventilation
FiO_2_	1.0	0.45
PaO_2_ (75–100 mmHg)	62.2 mmHg	102 mmHg
PaCO_2_ (38–42 mmHg)	50.9 mmHg	31.9 mmHg
Cardiac functions		
High sensitive troponin I (< 0.004 ng/ml)		3.45 ng/ml
Brain natriuretic peptide (BNP) (20 pg/ml)		1130 pg/ml
Other		
C-reactive protein (CRP) (< 6)	171 on (12th /10/2021)	77.5
Lactate dehydrogenase (LDH) (140–280 U/L)		12,104 U/L
Lactate (< 2.3 mmol/L)	7.6 mmol/L	8.1 mmol/L
pH (7.35–7.45)	7.13	7.26
Base excess (− 2 to − 2 mEq/L)	− 11.8 mEq/L	− 12.7 mEq/L

**Fig. 2:**
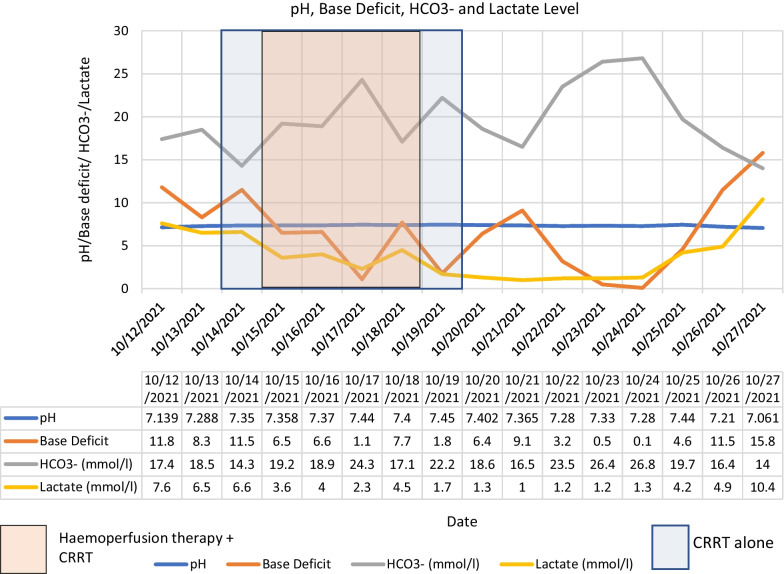
Progression of acid base balance and serum lactate levels during the ICU stay. Please note the correction of the metabolic acidosis with the use of CRRT and its reappearance after discontinuation of CRRT. Subsequently, the acidosis got corrected along with organ recovery until fungal sepsis ensued

**Fig. 3:**
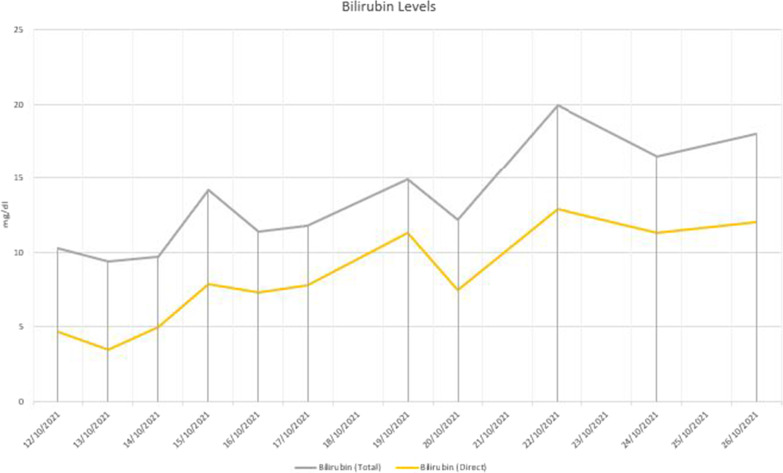
Progression of bilirubin levels during the ICU stay. The transaminase levels dropped progressively, but the bilirubin levels were rising. Thrombocytopenia and haemolysis persisted even after the coagulopathy was corrected

**Fig. 4:**
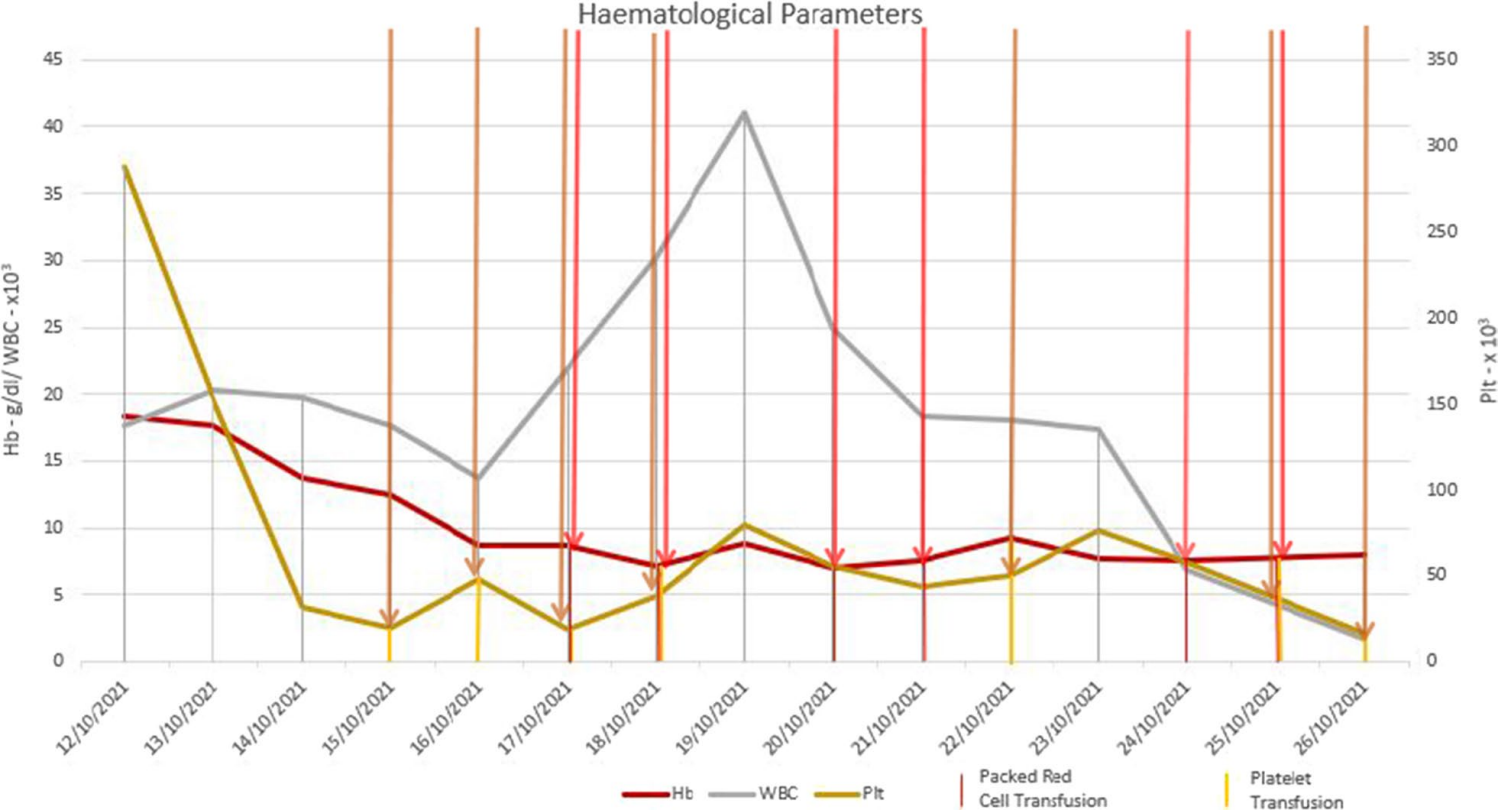
Progression of haematological parameters during the ICU stay. Blood and platelet transfusions are indicated by the red and yellow lines. The transaminase levels dropped progressively, but the bilirubin levels were rising. Thrombocytopenia and haemolysis persisted even after the coagulopathy was corrected

**Fig. 5:**
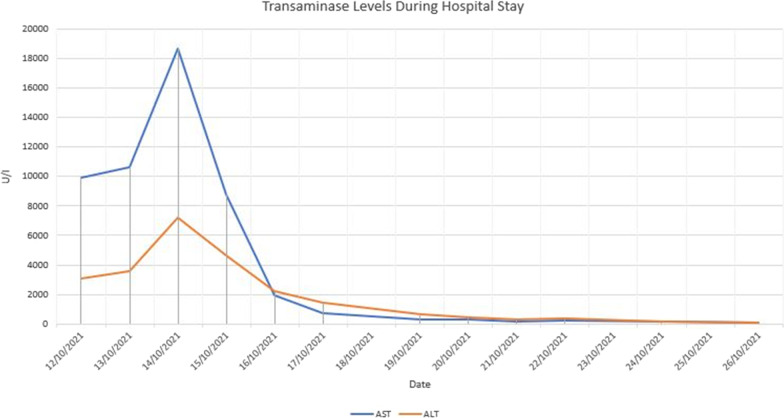
Progression of liver enzymes during the ICU stay. The transaminase levels dropped progressively, but the bilirubin levels were rising. Thrombocytopenia and haemolysis persisted even after the coagulopathy was corrected

**Fig. 6:**
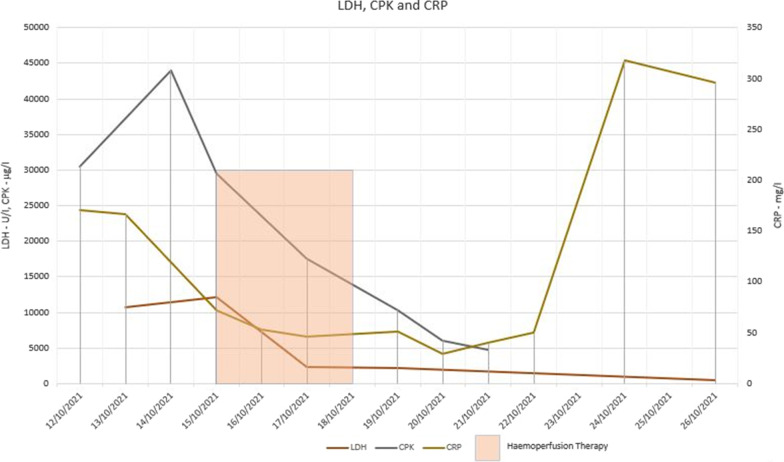
Progression of LDH, CPK, and CRP during the ICU stay. The transaminase levels dropped progressively, but the bilirubin levels were rising. Thrombocytopenia and haemolysis persisted even after the coagulopathy was corrected

**Fig. 7:**
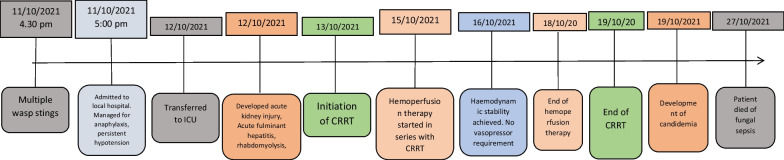
Timeline of events

He was initiated on continuous renal replacement therapy (CRRT—CVVHDF—continuous veno-venous haemodiafiltration) from the 3rd day due to progressive multi-organ failure, and continued till the 8th day. Despite initiating CRRT, his organ functions deteriorated with worsening lactic acidosis. Haemoadsorption therapy using extra-corporeal whole blood adsorber was used in series with CRRT from 4th to 7th days, with the intention of removing wasp toxins, myoglobin, bilirubin and cytokines (large molecular compounds). CRRT dose was escalated from 25 ml/kg/h to 35 ml/kg/h due to worsening metabolic acidosis. Fluctuation of his acid base status during the hospital stay is given in Fig. 2. The vasopressor requirement diminished gradually and was tailed off by the 5th day. Urine production gradually increased from the 4th day. The fluctuation of biochemical and haematological parameters during the hospital stay is given in Figs. 3, 4, 5, 6 and the timeline of events in Fig. 7.

From the 7th day of admission, blood-cultures were persistently positive for *Candida* which was treated with IV fluconazole (echinocandins were not available in the low-resource setting, the patient was managed). The patient’s clinical parameters started to deteriorate from day 12, with increasing vasopressor requirement. The patient passed away on the 16th day due to overwhelming fungal sepsis despite increasing organ support.

## Discussion

Wasp stings can result in a spectrum of clinical conditions, ranging from mild local reactions to fatal systemic reactions with multi-organ dysfunction. This case report of multi-organ dysfunction, following wasp stinging acts as a primer to study the various clinical manifestations of wasp envenomation and its underlying pathophysiology. The organ injury is either secondary to anaphylactic shock or due to the direct effect of the toxins.

Anaphylaxis, an IgE-mediated immediate hypersensitivity reaction with mast cell degranulation, resulting in generalized vasodilatation and organ hypoperfusion is the most recognized systemic effect of wasp stings. Due to its immune-mediated pathogenesis, anaphylaxis is more common in patients who have been exposed to the venom previously, in patients with a known allergy to insects and in patients with background of atopy [3]. However, not all circulatory collapse following wasp stinging is due to anaphylaxis and could be secondary to the direct effect of toxins on the circulatory system [4].

Toxin-mediated organ injury can result in AKI, acute liver injury (ALI) with or without fulminant hepatic failure, haemolysis, disseminated intravascular coagulation (DIC), rhabdomyolysis, cardiac injury and acute respiratory distress syndrome or pulmonary haemorrhage. These organ injuries are heralded by a systemic hyper-inflammatory state, a cytokine storm syndrome [4].

Wasp stings can have delayed manifestations, such as interstitial nephritis [5], encephalopathy [6], vasculitis and is postulated to be driven by the activation of the immune system leading to hypersensitivity or autoimmunity.

### Wasp venom components

During a sting, the wasp injects the proteinaceous liquid stored in its venom sac, i.e., the wasp venom. The venom constitutes of enzymes, amines, peptides and other compounds [7]. A proteomic study of the wasp *Vespa affinis* venom revealed 93 protein spots, of which proteins in 51 spots had been identified and classified into three groups: typical venom components, structural and housekeeping proteins. Thirty-two venom proteins had reacted with IgE of wasp allergic patients indicating the potential for allergy [8].

#### Enzymes

The two main enzymes found in wasp venom are phospholipases and hyaluronidase. Phospholipase A (PLA) a calcium-dependent enzyme is further categorized into PLA 1 and PLA 2, both of which act on the cell membranes, hydrolyse the phospholipids and lead to cell lysis, including red blood cells. The by-products of the hydrolysis, such as lysophosphatidylcholine, lysophosphatidic acid and sphingosine 1-phosphate, can have cytotoxic and immunostimulatory effects on diverse cell types, causing inflammation and activation of the immune system [9, 10]. Furthermore, PLA acts as an antigen, binds to IgE and results in allergic and anaphylactic reactions. PLA is found in all *Hymenoptera* species while phospholipase A2 (PLA2) is a predominant component of bee venoms, phospholipase A1 (PLA1) is highly abundant in wasps and ants [11].

Hyaluronidase lyses the extracellular matrix by breaking the hyaluronic acid into non-viscous fragments, aiding in the local spread of the venom and entering blood vessels, resulting in systemic envenomation. Products of hyaluronan degradation are pro-inflammatory, pro-angiogenic and immunostimulatory [9]. Other enzymes found in wasp venom are phospholipase B, phosphatases and alpha-glucosidases.

#### Amines

The wasp venom contains the amines; histamine, serotonin and several catecholamines among many others. The histamines increase vascular permeability and enhance cytolytic activity. Serotonin causes vasospasms and along with histamine stimulates peripheral nociceptors to cause pain [12]. Catecholamines cause tachycardia and aid in the spread of the venom once it reaches the circulation. However, the effects of histamines and catecholamines are largely overshadowed by the effects of the other toxins [9].

#### Peptides

There are numerous peptides in the wasp venom. Mastoparans, a group of multi-functional toxins are membrane-active amphipathic peptides which insert into the plasma membrane bilayer causing membrane destabilization and cell lysis. As the name implies it can cause mast cell degranulation releasing histamine, along with degranulation of chromaffin cells, platelets, anterior pituitary and pancreatic beta cells releasing catecholamines, serotonin, prolactin and insulin, respectively [13]. Furthermore, it can trigger cell apoptosis by interacting with the cytoplasmic subunits of G-protein-coupled receptors and by activation of second messengers leading to Ca^2+^ mobilization from the mitochondria and sarcoplasmic reticulum [9, 14, 15]. Mastoparans have also been shown to inhibit the Na^+^/K^+^ ATPase of the renal tubular cells in rats and might be involved in the pathogenesis of acute tubular necrosis following wasp stings [16].

Melittin, found mainly in bees, can also be found in wasps. It is a weak allergen, but induces pain by direct and indirect actions on primary nociceptor cells. Melittin can form pores in cell membranes causing cell lysis and activating the inflammatory response [17].

Apamin, another peptide in wasp venom, can selectively inhibit Ca^2+^-dependent K^+^ channels in the central nervous system and results in continuous firing of neurons in the mesencephalon and cerebellum, elevating cell sensitivity to excitatory inputs. Conversely, apamin inhibits the transmission at the neuromuscular level [17].

Wasp kinins are structurally and chemically similar to bradykinin. They increase vascular permeability and cause oedema. Furthermore, they can cause pain and are weak mast cell degranulators [18].

Some other toxins found in wasp venom are [9]:

1. Mast cell degranulating peptide—has anti-inflammatory effects at high concentrations,
2. Adolapin—an anti-inflammatory peptide,
3. Tertiapin—an inward rectifying potassium channel blocker and
4. Scapins induce leukotriene-mediated hyperalgesia and oedema.

There is ongoing research to utilize proteins found in wasp venom in antimicrobial and anti-neoplastic therapeutics [19].

#### Other components

Other components of wasp venom, such as acetylcholine can cause intense depolarization of the nociceptors within the dermis and result in pain. Antigen 5, a glycosylated allergenic protein induces a strong acute hypersensitivity response [20], [20]. Mortality following wasp stings increases with the venom dose injected, as reflected by the number of stings [4].

### Wasp venom effects

#### Anaphylaxis

The first reported human anaphylactic death is considered to be the death of Pharaoh Menes, caused by a wasp sting [22]. Anaphylactic shock is the major mechanism of mortality and organ injury following wasp stings [23]. PLA, hyaluronidase, and antigen 5 are known to cause IgE-mediated type 1 hypersensitivity reactions. In a Chinese multi-centre retrospective study with 1091 patients, 22% were diagnosed with anaphylactic shock. A similar proportion presented with shock which was not due to anaphylaxis [4]. Non-anaphylactic shock could be due to toxin-mediated mast cell degranulation and direct vasodilatory and cardioinhibitory actions of the toxins. This is further aggravated and maintained by the systemic inflammatory hyperactivation.

In the above Chinese study, 25 patients were tested for venom-specific IgE and venom-reactive lymphocyte subsets. Of them, 16 had venom-specific IgE to at least 1 of 7 common wasp venom proteins, but surprisingly none of them had developed anaphylactic shock or cardiac arrest, although toxin-mediated organ injury was observed in them [4]. Therefore, the presence of wasp venom-specific IgE alone might not be indicative of developing anaphylaxis following a wasp sting.

#### Hyper-inflammatory response and multi-system involvement

Wasp envenomation is known to cause systemic hyper-inflammatory reaction. Several studies have demonstrated elevated cytokine levels including Interleukin-2 (IL-2), IL-6, IL-8, IL-10, IL-17, Interferon-γ (IFN-γ), and tumour necrosis factor-α (TNF-α) following wasp stinging [4, 7, 24]. The cytokine levels correlated positively with the white blood cell counts, serum creatinine and the C-reactive protein levels [24]. Whole transcriptome analysis following wasp stinging demonstrated evidence of highly expressed pro-inflammatory responses, including leucocyte activation, neutrophilic degranulation, and humoral immune responses [25]. The immune activation results in a systemic inflammatory response leading to multi-organ dysfunction.

#### Renal effects

The incidence of AKI from wasp envenomation ranges from 10 to 58% [7, 26, 27]. The largest case series with 1091 patients from China showed an incidence of 21.0% [4]. Out of the patients who developed kidney injury, more than 80% had severe AKI and required dialysis [28–30]. The variation could be due to the difference in wasp species and health seeking behaviours of the people in different geographies [31].

The AKI is characteristically oliguric (70%) [26, 32]. Urinalysis usually shows various amounts of red and white blood cells with or without casts. A minority can present with gross haematuria [4, 26]. There are case reports where heavy protein on dipstick was detected [25].

The pathophysiology of the kidney injury is commonly due to; acute tubular necrosis (ATN), effects of myoglobin and haemoglobin on the kidneys, effects on renal perfusion and tubulo-interstitial nephritis (AIN) or any combination of the above. In a case series with 11 patients who developed AKI following wasp stings, renal biopsy was done on four patients, AIN was seen in one patient, ATN in two patients, and one patient had both AIN and ATN [32].

ATN results from direct nephrotoxic molecules in the wasp venom, renal ischaemia due to hypotension and microvascular circulatory dysfunction. The ability of PLA and mastoparan to disrupt renal cell membranes and cause cell necrosis is a contributing factor for ATN [33]. Nephrotoxicity of the wasp venom was demonstrated in animal studies [34]. Renal biopsies have shown evidence of isolated tubular cell necrosis, [35] although haemoglobin and myoglobin pigment casts are usually seen in addition [25]. Haemolysis and rhabdomyolysis release haemoglobin and myoglobin into the blood. Haemoglobin and myoglobin can cause acute kidney injury by direct toxic effects on the tubular cells, renal vasoconstriction and tubular obstruction by cast formation [36].

Anaphylactic or non-anaphylactic circulatory shock following wasp stings can lead to relative hypovolaemia. Furthermore, rhabdomyolysis can cause leakage of plasma into damaged myocytes causing an absolute hypovolemia in the intravascular compartment [36]. Moreover, there is microcirculatory dysfunction during the systemic inflammatory response of the body. All this could lead to renal hypoperfusion.

Tubulointerstitial nephritis (AIN) is another common mechanism of AKI following wasp stings. Numerous molecules in wasp venom that cause AIN have been identified. Evidence for the presence of AIN stems from renal biopsies and response to steroids [37]. Renal biopsies in AIN following a wasp sting are characterized by; normal glomeruli, peritubular eosinophilic and mononuclear cell infiltration, with hyaline and pigmented granular casts in the tubules [38–40]. Combinations of the above pathologies are a common finding in renal biopsies. AIN with pigment casts [41], ATN and AIN [42] are such examples.

Rare mechanisms of kidney injury, such as, renal cortical necrosis [43] and thrombotic microangiopathy [44] have been documented. Although exceptionally rare, nephrotic syndrome [45] with minimal change [46] and mesangial-proliferative glomerulonephritis [47] have been reported following wasp stings.

A transcriptome analysis of the kidney done after 2 weeks of developing AKI following wasp stinging demonstrated increased expression of *SULF2, JCHAIN,* and *PARKAR2B.* Although the implications of these gene expressions are not clear, they might reflect the regeneration of the renal cells during recovery [25].

Yuan et al. [48] in a retrospective cohort study of 112 participants, compared patients with and without AKI and identified elevated leukocytes (> 10 × 10^9^/L) [OR 1.12 (95% CI 1.02–1.23)], high myoglobin (> 1200 ng/mL) [OR 18.51 (95% CI 1.51–132.27)], and high urinary monocyte chemotactic protein-1 (MCP-1) (> 200 pg/mL) [OR 5.42 (95% CI 1.27–30.39)] as independent predictors of development of AKI following wasp stings. A Vietnamese study with 65 patients demonstrated that the higher the number of wasp stings the greater the chance of developing AKI [27]. The levels of interleukin (IL) -6, IL-10, and IL-17 positively correlated with the risk of developing AKI [24]. The elderly [49] and patients with macroscopic haematuria had a severe course of illness [26].

Majority of the patients who develop AKI following wasp stings recover completely with an average recovery time of 36 days [50]. However, around 10% of patients were found to progress in to end stage kidney disease (ESKD) [48, 50]. A higher level of ALT, AST, LDH, CPK-mb, APTT, PT, and proteinuria on admission were associated with progression to ESKD. The underlying pathology in patients progressing to ESKD is not known.

The index patient developed acute kidney injury within 24 h of the wasp sting. We could not perform a renal biopsy to conclude the exact pathology of the AKI due to his critical illness and coagulopathy. However, evidence of early renal recovery was observed with the improvement in the urine output until the secondary fungal sepsis resulted in a second episode of AKI.

#### Hepatic effects

The incidence of hepatotoxicity following wasp stinging is around 30% [4, 27]. Hepatitis syndrome is the commonest pattern among patients who develop liver injury, with disproportionate rise in transaminases compared to the elevation of alkaline phosphatase and gamma glutamyl transferase [4]. Identification of the hepatic injury can be challenging as the elevation of transaminases is supplemented by the concurrent rhabdomyolysis, haemolysis and cardiac injury. Haemolysis causes a rise in indirect bilirubin levels and the presence of direct, mixed hyperbilirubinemia points towards a hepatic injury. A decrease in hepatic synthetic functions can also be observed, although the coagulopathy is contributed by DIC. A significant proportion will progress to acute fulminant hepatic failure [51] while other patterns of hepatic injury such as Reye syndrome have also been observed [52].

The pathogenesis of hepatic injury due to wasp stinging has not been studied extensively as for the kidneys in humans. There are case reports where centrilobular necrosis was observed in liver biopsies following wasp stings [53, 54]. Studies where animals were experimentally injected with wasp venom, demonstrated a significant elevation of liver enzymes along with markers of Kupffer cell damage. Similar observations were made when the perfusion of the isolated, intact, rat liver in situ, while measuring the liver enzyme leakage into the perfusate and bile [55, 56]. Light microscopy and histochemistry showed foci of hepatic necrosis, a decrease in glycogen content and in succinic dehydrogenase activity, some fat infiltration and an increase in alkaline phosphatase activity. Electron microscopy showed; a decrease in the number of mitochondria per cell, a decrease in their cristae, enlargement of bile canaliculi, and destruction of the endothelium of the sinusoids bringing the cytoplasm of the necrotic hepatocytes in contact with the lumen of the sinusoid [57].

Melittin stimulates glycogenolysis and induces vasoconstriction in perfused rat liver. The effect was rapid and associated with the production and release of prostaglandin D2 and thromboxane B2 [58]. However, mastoparan-1 has been shown to protect hepatocytes from lipopolysaccharide induced damage in animal studies [59].

The index patient developed acute fulminant hepatic failure within 24 h of the wasp sting. The liver transaminase levels and the lactic acidosis markedly improved with haemoperfusion therapy. Coagulopathy normalized by the end of the 13^th^ day since admission. However, the bilirubin levels continued to increase. The final outcome of the liver injury could not be assessed as the patient succumbed to secondary sepsis.

#### Effect on skeletal muscles

In the Chinese study with 1091 cases, elevated CPK was found in 53.7% of patients whereas 24.1% were diagnosed with rhabdomyolysis [4]. Similar rates have been observed in other series [26]. Although rare, myonecrosis has also been observed following wasp stings [60].

Rhabdomyolysis is attributed to skeletal muscle cell membrane disruption by PLA1 and melittin in wasp venom [61]. Mastoparan can induce myonecrosis, apoptosis and cytokine activation [62]. There are probably many other molecules and mechanisms contributing.

Patients commonly present with weakness, myalgia and muscle swelling. There can be associated skin necrosis and is considered a poor prognostic sign [63]. As expected, patients who develop rhabdomyolysis have a higher incidence of AKI [4]. There are case reports where forced alkaline diuresis was used to prevent rhabdomyolysis induced AKI following wasp stings [64].

#### Haematological effects

Most patients who suffer wasp stings have a reactive neutrophil leucocytosis. However, the pathological consequences are haemolysis, thrombocytopenia, coagulopathy and DIC.

The incidence of haemolysis following a wasp sting ranges from 17 to 22% [4, 26]. As mentioned previously, the mechanism of red cell lysis is disruption of the cell membrane by phospholipases and other toxins found in wasp venom [9]. Haemolysis has been noted even after a single wasp sting in children [65, 66].

A prolonged APTT, reduced factor Xa activity and low fibrinogen levels following wasp stings have been noted by many, even in the absence of DIC [67]. The first two observations were attributed to heparin release from mast cells and the latter due to the effect of tryptase on fibrinogen and its ability to activate plasminogen [68, 69]. This phenomenon was named endogenous hyper-heparinization. This theory was further strengthened with the partial reversal of coagulopathy in thromboelastographic studies when protamine sulphate or heparinases were used [70]. The coagulopathy seemed to correlate with the wasp venom dose [27].

It was later found that the wasp venom itself has anti-coagulant effects. The venom seemed to be affecting various steps of intrinsic, extrinsic and common pathways and demonstrated fibrinogen degrading properties [71]. Subsequently, a serine protease named magnvesin was isolated from the venom of *Vespa magnifica.* Magnvesin exerts its anti-coagulant function by hydrolysing tissue factor and the clotting factors, VII, VIII, IX and X [72]. Furthermore, wasp venom can inhibit platelet aggregation [71] and can cause thrombocytopenia even in the absence of DIC [53].

#### Cardio-vascular effects

It was discussed previously that shock following wasp stings could be anaphylactic or due to direct vasodilatory effects of the toxins or both. The patients with a persistent shock beyond the anaphylactic period are known to have a higher mortality [27].

Myocardial injury has been observed following wasp stings. Three mechanisms of myocardial injury have been identified. Them being, myocardial infarction, hypersensitivity myocarditis, and Takotsubo cardiomyopathy [73]. Myocardial infarction following wasp stings [74, 75] is caused by either hypotension due to shock or coronary vasoconstriction, which is induced by the released chemical mediators (histamine, chymase, tryptase, cathepsin D, leukotrienes, thromboxane and platelet activating factor) [22] and treatment with epinephrin. Many angiographic studies have demonstrated coronary artery vasospasms. Thrombosis with or without [76] disruption of pre-existing atherosclerotic plaques is another mechanism [77]. Thrombosis of coronary stents has also been noted [78]. Acute coronary syndromes associated with anaphylaxis are also known as Kounis syndrome and is extensively described following wasp stings [79]. Cardiac MRI in such instances will show enhancement in the affected myocardium [80]. A case of silent ST elevated myocardial infarction was reported in a patient without autonomic neuropathy or coronary atherosclerosis [81]. Whether this was due to the toxin’s effect on cardiac nerves is not known.

Takotsubo cardiomyopathy or stress cardiomyopathy, occurs due to a shift of fat metabolism to glucose metabolism in cardiomyocytes during a catecholamine surge. Patients have ST elevations in the ECG, positive cardiac biomarkers and left ventricular apical ballooning without significant coronary artery stenosis. Takotsubo cardiomyopathy has been described following wasp stings [82]. Although, identified as an early feature there are cases of delayed onset Takotsubo cardiomyopathy following wasp stings [83].

Direct cardiotoxic effects of the wasp venom have been postulated. Animal studies using Wistar rats demonstrated necrosis of the myocardium; and enzyme histochemistry showed inactivation of enzymes in and around the areas of necrosis [84]. Some patients, classified as having ischaemic or stress induced cardiac damage might also be having direct cardiotoxic damage due to the wasp venom. Furthermore, atrial fibrillation, flutter and many other arrhythmias have been noted following wasp stings [85, 86]. Please write our patients findings.

#### Nervous system effects

The nervous system manifestations following wasp stings can be classified as early and delayed. Early manifestations are due to the hypotension, anaphylaxis or direct neurotoxicity of the wasp venom. Delayed manifestations appear to be immune-mediated hypersensitive reactions.

Post mortems of patients who died of wasp stings, revealed cerebral oedema, petechial haemorrhages, congestion of cerebral and pial vessels, and encephalomalacia. These features are non-specific and have been observed following anaphylaxis [87]. Ischaemic strokes have occurred following wasp stings and the pathophysiology is considered to be similar to that of Kounis syndrome [88, 89].

Certain wasps can paralyse their prey following a sting indicating that there are neurotoxins in their venom in addition to what’s described above. Pompilidotoxins in the venoms of the pompilid wasps can slow Na^+^ channel inactivation in nerves [18]. Emerald jewel wasps (*Ampulex compressa*) zombify the American cockroach (*Periplaneta americana*) with a sting to the brain. When the venom takes effect, the cockroach becomes passive and can be led by its antenna into a hole, where the wasp deposits an egg and then seals the exit with debris. The cockroach has the ability to walk, run, or fly if properly stimulated, but it does not try to escape as it is slowly eaten alive by the developing wasp larva [90]. Insects have a myriad of interesting neurotoxins [91], some of which are being researched to be used as anti-seizure medication [92] and against neurodegenerative diseases [93].

The delayed nervous system manifestations following wasp stings are diseases with an inflammatory pathology. Such manifestations are given in Table 2 [6].

**Table 2:** Neurological manifestations following wasp stings

Central nervous system	Peripheral nervous system
Acute encephalopathy [94, 95]	Guillain–Barre syndrome[96]
Encephalopathy with extrapyramidal symptoms [95]	Miller Fisher syndrome
Coma with catatonia [95]	Trigeminal neuralgia [97]
Acute disseminated encephalomyelitis [98]	Autoimmune Neuromyotonia [99]
Posterior reversible encephalopathy syndrome [100]	Myasthenia gravis [101]

In the case of neuromyotonia (diagnosed with a nerve conduction study) antibodies to the voltage gated potassium channel (VGKC) in conjunction with a wasp venom-specific immunoglobulin E (IgE) were detected. It had responded only to plasma exchange where levels of both VGKC antibodies and total IgE had fallen in parallel with the patient's clinical recovery.

Although included in the above table, due to its immune-mediated pathophysiology, the case of myasthenia gravis occurred within 24 h. It could have been due to an immediate hypersensitivity reaction to some components of wasp venom or a direct toxic effect of these substances on acetylcholine synthesis, release, or degradation.

#### Other system involvement

The other clinical features of systemic envenomation of wasp toxins are given in Table 3.

**Table 3:** Other organ system effects of wasp stinging

Organ system	Manifestation
Respiratory system	Diffuse alveolar haemorrhage [102, 103] Pleural effusions [104] Acute pulmonary oedema [98, 99] Acute respiratory distress syndrome [25]
Gastro-intestinal	Fatal GI bleeding [106] Non-occlusive mesenteric ischaemia [107] Pancreatitis [108] Spontaneous splenic rupture [109]
Rheumatological	Henoch–Schönlein purpura [110]
Skin	Skin necrosis (biopsy of skin—evidence of vasculitis)[111] Skin haemorrhage [112]
Ocular	Corneal decompensation [113] Cataracts [113]

### Mortality

The mortality following wasp envenomation ranges from 4 to 10% [26, 50]. The mortality from organ dysfunction was higher than that due to anaphylaxis. In the series with 1091 patients, six died of anaphylaxis while 48 died of organ injury “following toxic reactions to the stings” [4]. Even among patients who developed shock, the morality of the non-anaphylactic group (44%) was higher than that of the anaphylactic shock (25%) group [4].

Mortality increased with increasing number of stings [4].The presence of an organ injury significantly increased the mortality rate. In a series of 55 patients, 36.4% died due to multi-organ failure, where the kidney and the liver were the most involved organs. Absence of an AKI acts as a good prognostic factor; in a retrospective cohort study with 112 patients, there were no deaths in the group who didn’t develop AKI [48]. A high creatinine level, development of shock, oliguria, and anaemia were found to be predictors of increased mortality [48].

### Treatment

There are no established guidelines on the management of wasp envenomation. Providing a complete therapeutic guidance is beyond the scope of this review. Although there is no trial evidence, antihistamines and topical or oral corticosteroids are used to treat local reactions following wasp stings; and is endorsed by the NHS as a first aid measure [114]. A trial with 15 patients revealed that pre-medication with leukotriene antagonists reduced the local reactions in allergen immunotherapy. However, whether this can be translated to clinical practice is not known [115].

The section below is an outline of the management strategies used in various case studies for systemic envenomation.

Management of a systemic wasp envenomation falls into three main steps:

1. Management of anaphylaxis.
2. Prevention of organ injury.
3. Provision of organ support.

Management of anaphylaxis is straightforward and will not be discussed here. Maintaining tissue perfusion is a cardinal step in prevention of organ injury. Fluids and ionopressors should be used to maintain adequate organ perfusion pressure. Dehydration can hasten pigment cast formation in the kidneys contributing to development of AKI. Attention should be paid to preventing/treating electrolyte imbalances.

The next important step in preventing organ injury is the removal of venom toxins and toxic substances released from organ damage, such as myoglobin and haemoglobin. Forced alkaline diuresis has been used for the removal of haem-proteins and was found to prevent the need for dialysis in some small studies [106]. However, this might not always be possible and it mandates the presence of a urine output, hence, is not useful if oliguria has set in.

Removal of venom and endogenous toxins cannot be done using simple renal replacement therapy as they are high molecular weight compounds but can be improved by combining with adsorbent therapies such as haemo-perfusion. High volume haemofiltration has been shown to be more efficacious than intermittent haemodialysis alone [116].

Haemoperfusion uses an adsorbent charcoal cartridge or an ion exchange resin and is connected in series with the CRRT. Endotoxins, superantigens, cytokines and certain agents that are not removed by dialysis or haemofiltration get adsorbed to the material in the cartridge.

Plasma exchange (PE) has also been used to treat multi-organ dysfunction following wasp envenomation. However, plasma exchange alone has not been shown to be effective whereas PE combined with haemo-perfusion has proven to be efficacious [117]. Although, there might be a place for immunosuppression to prevent delayed immune-mediated organ injury in selected cases, there is no evidence for the routine use of steroids in wasp envenomation; as this can increase the risk of secondary infection. There is no evidence for use of pooled human immunoglobulins (IVIG) in the treatment either. The only recorded cases where IVIG has been used are, to treat immune thrombocytopenic purpura developed after bee venom exposure [118].

Moreover, extra-corporeal membrane oxygenation (ECMO) has been used in the event of pulmonary haemorrhage following wasp stings [103].

### Limitation

Most of the studies used in this review have not identified the species of the wasp. Whether there are species-specific differences in wasp venom toxicity are not known.

## Conclusion

Wasp venom constitutes a myriad of proteins which can affect many organ systems of the body. The effects of wasp envenomation could be due to immediate hypersensitivity reactions (anaphylaxis), direct toxin-mediated cell injury and associated systemic inflammation or delayed immune reactions. The main organ systems affected by wasp venom include the kidneys, liver, blood cells, skeletal muscles and the cardiovascular system. Neurological manifestations are rare and mostly arise as type II or III hypersensitivity reactions. Involvement of other organ systems is seen occasionally. Treatment of wasp envenomation is mainly supportive although immunomodulatory therapies, such as haemoperfusion and plasma exchange have been used successfully.

## Data Availability

A copy of the clinical records of the patient is available with the authors. The original records are stored in the record room National Hospital Sri Lanka. The authors are willing to share the available clinical records if needed.

## Abbreviations

AIN: Acute tubulo-interstitial nephritis
AKI: Acute kidney injury
ALI: Acute liver injury
ALP: Alkaline phosphatase
ALT: Alanine transaminase
APTT: Activated partial thromboplastin test
AST: Aspartate transaminase
ATN: Acute tubular necrosis
BNP: Brain natriuretic peptide
CPK: Creatine phosphokinase
CRP: C-reactive protein
CRRT: Continuous renal replacement therapy
CVVHDF: Continuous veno-venous haemodiafiltration
DIC: Disseminated intravascular coagulation
ECMO: Extra-corporeal membrane oxygenation
ESKD: End stage kidney disease
FiO2: Fraction of inspired oxygen
Hb: Haemoglobin
ICU: Intensive care unit
IFN-γ: Interferon-γ
IgE: Immunoglobulin E
IL: Interleukin
IM: Intra-muscular
INR: International normalized ratio
IV: Intra-venous
IVIG: Pooled human immunoglobulins
LDH: Lactate dehydrogenase
MCP-1: Monocyte chemotactic protein-1
PaCO2: Partial pressure of arterial carbon dioxide
PaO2: Partial pressure of arterial oxygen
PLA: Phospholipase A
PE: Plasma exchange
Plt: Platelet
PT: Prothrombin time
S/Cr: Serum creatinine
S/K +: Serum potassium
S/Na +: Serum sodium
TNF-α: Tumour necrosis factor-α
WBC: White blood cells
